# Design and Rationale of the APPELHUS Phase 3 Open-Label Study of Factor B Inhibitor Iptacopan for Atypical Hemolytic Uremic Syndrome

**DOI:** 10.1016/j.ekir.2023.04.029

**Published:** 2023-04-29

**Authors:** David Kavanagh, Larry A. Greenbaum, Arvind Bagga, Rajeshri G. Karki, Chien-Wei Chen, Sajita Vasudevan, Alan Charney, Marion Dahlke, Fadi Fakhouri

**Affiliations:** 1 National Renal Complement Therapeutics Centre, Newcastle upon Tyne Hospitals NHS Foundation Trust, Newcastle upon Tyne, UK, and Complement Therapeutics Research Group, Translational and Clinical Research Institute, Newcastle University, Newcastle upon Tyne, UK; 2 Division of Pediatric Nephrology, Emory School of Medicine and Children’s Healthcare of Atlanta, Atlanta, Georgia, USA; 3 Department of Pediatrics, All India Institute of Medical Sciences, New Delhi, India; 4 Clinical Development and Analytics Group, Cardiovascular, Renal and Metabolism Development Unit, Novartis Pharma, East Hanover, New Jersey, USA; 5 Chief Medical Office and Patient Safety, Novartis Healthcare, Hyderabad, India; 6 Clinical Development and Analytics Group, Cardiovascular, Renal and Metabolism Development Unit, Novartis Pharma, Basel, Switzerland; 7 Centre Hospitalier Universitaire Vaudois (CHUV), Lausanne, Switzerland

**Keywords:** LNP023, aHUS, alternative pathway, atypical hemolytic uremic syndrome, Factor B, iptacopan

## Abstract

**Introduction:**

Atypical hemolytic uremic syndrome (aHUS) is a rare, progressive, and life-threatening form of thrombotic microangiopathy (TMA) which is caused by dysregulation of the alternative complement pathway (AP). Complement inhibition is an effective therapeutic strategy in aHUS, though current therapies require intravenous administration and increase the risk of infection by encapsulated organisms, including meningococcal infection. Further studies are required to define the optimal duration of existing therapies, and to identify new agents that are convenient for long-term administration. Iptacopan (LNP023) is an oral, first-in-class, highly potent, proximal AP inhibitor that specifically binds factor B (FB). In phase 2 studies of IgA nephropathy, paroxysmal nocturnal hemoglobinuria, and C3 glomerulopathy, iptacopan inhibited the AP, showed clinically relevant benefits, and was well tolerated. Iptacopan thus has the potential to become an effective and safe treatment for aHUS, with the convenience of oral administration.

**Methods:**

Alternative Pathway Phase III to Evaluate LNP023 in aHUS (APPELHUS; NCT04889430) is a multicenter, single-arm, open-label, phase 3 study to evaluate the efficacy and safety of iptacopan in patients (*N* = 50) with primary complement-mediated aHUS naïve to complement inhibitor therapy (including anti-C5). Eligible patients must have evidence of TMA (platelet count <150 × 10^9^/l, lactate dehydrogenase ≥1.5 × upper limit of normal, hemoglobin ≤ lower limit of normal, serum creatinine ≥ upper limit of normal) and will receive iptacopan 200 mg twice daily. The primary objective is to assess the proportion of patients achieving complete TMA response without the use of plasma exchange or infusion or anti-C5 antibody during 26 weeks of iptacopan treatment.

**Conclusion:**

APPELHUS will determine if iptacopan is safe and efficacious in patients with aHUS.

## Graphical abstract

**Figure:**
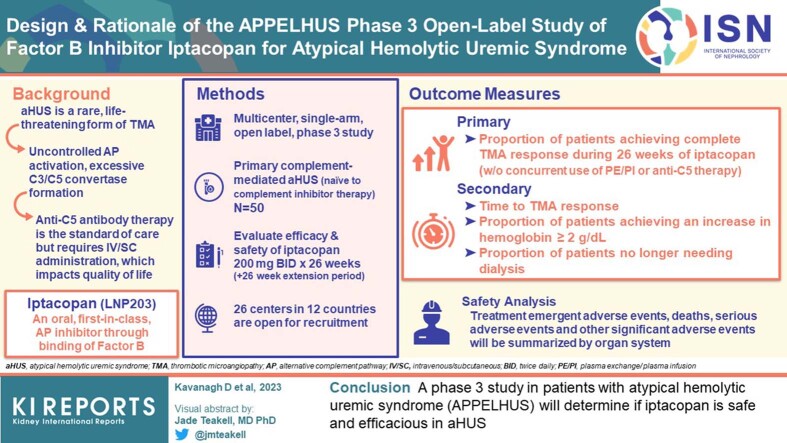

aHUS is a rare, progressive, life-threatening form of TMA characterized by microangiopathic hemolytic anemia, thrombocytopenia, and acute kidney failure.^1^, ^2^, ^3^, ^4^ There is limited information on worldwide epidemiology; however, the literature reports incidence as 0.5 to 2/million/yr.^1^^,^^5^ Patients with aHUS have a poor prognosis before the availability of eculizumab, an anti-C5 antibody therapy. In the pre-eculizumab era, approximately 36% to 48% of children and 64% to 67% of adults reached kidney failure and/or death by 3 to 5 years after onset.^6^, ^7^, ^8^

There have been significant advances in our understanding of the underlying pathophysiology of aHUS,^6^ and we now know that uncontrolled activation of the AP is the pathogenic mechanism in most cases of the disease.^4^ The AP is normally constitutively active at low levels and plays an important role in host defense by amplifying the complement response to pathogens.^9^^,^^10^ Ordinarily, activation of the AP is tightly regulated;^7^ however, in aHUS dysregulation results in excessive formation of C3 and C5 convertases and consequent formation of the membrane attack complex (MAC) on the vascular cells mainly in the kidneys, which is key for the disease phenotype, leading to endothelial cell injury, cell detachment and, ultimately, a thrombotic state: thrombus formation, platelet consumption, vascular occlusion, and mechanical hemolysis^2^ (see Figure 1 for further information). In studies of patients with aHUS, approximately 60% have genetic or acquired abnormalities that impair the normal downregulation of the AP.^1^^,^^2^^,^^15^^,^^16^

**Figure 1:**
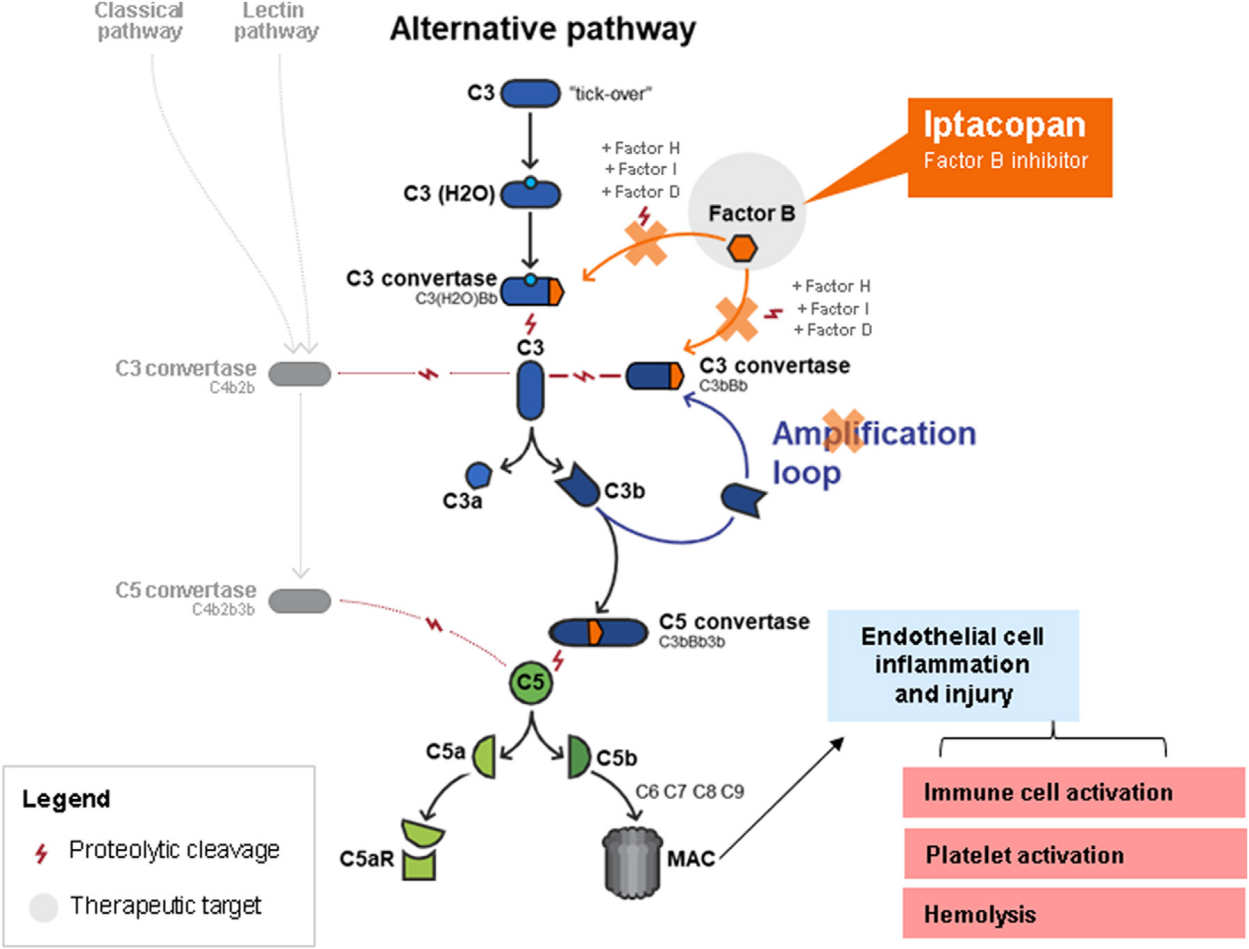
Iptacopan inhibits activation of the alternative pathway. In complement-mediated aHUS, dysregulation results in excessive formation of C3 and C5 convertases and consequent formation of the membrane attack complex, leading to endothelial cell injury, cell detachment and, ultimately, a thrombotic state: thrombus formation, platelet consumption, vascular occlusion, and mechanical hemolysis.^2^^,^^11^ Iptacopan does not inhibit the activation of the lectin and classical pathways, nor does it inhibit opsonization, formation of C3/C5 convertase, or membrane attack complex via these pathways.^12^, ^13^, ^14^ Iptacopan binds to FB to prevent activity of alternative complement pathway C3 convertases, inhibiting signaling from the alternative complement pathway and activation of the amplification loop. This prevents downstream generation of the alternative complement pathway C5 convertase complex, alternative complement pathway-dependent opsonization, and alternative complement pathway-mediated formation of C3a and C5a anaphylatoxins and membrane attack complex.^13^^,^^14^

The importance of the AP in the pathogenesis of aHUS was confirmed by the dramatic effectiveness of anti-C5 antibody therapy, which decreased the risk of patients developing kidney failure from approximately 70% to 15%.^17^^,^^18^ Therefore, the anti-C5 monoclonal antibodies eculizumab and ravulizumab have become the standard of care (SoC), along with supportive therapy.^18^

Despite the availability of effective treatment, patients may not have optimal quality of life because of residual kidney damage, financial worries given the high cost of therapy, concerns about the risk of infection, and the burden of therapy.^19^ Specifically, current SoC increases the risk of meningococcal infection^20^, ^21^, ^22^ and requires intravenous infusions every 2 to 8 weeks of eculizumab and ravulizumab, respectively, or weekly subcutaneous administration of ravulizumab. Injection site reaction is another concern and, moreover, these treatments are not available in all countries.^23^ With the rarity of the disease and limited specialist centers for treatment of aHUS, this burden is often compounded by the long distances patients need to travel to receive their recurring therapeutic infusions. Therefore, new targeted oral therapies have the potential to address some of the current limitations and will also create treatment choice.

aHUS is an acute disease with many patients presenting with nonspecific clinical symptoms, including fatigue, pallor, shortness of breath, and reduced urine output with or without edema.^2^, ^3^, ^4^ This varying clinical presentation, combined with the absence of diagnostic biomarkers for aHUS leads to difficulty in diagnosing complement-mediated aHUS from other causes of TMA and definitive test results (autoantibodies or genetic testing), which are only positive in approximately 60% of patients, are not typically available for days to weeks after clinical presentation. Therefore, a diagnosis of aHUS remains a clinical one relying on ruling out other forms of TMA,^16^ notably thrombotic thrombocytopenic purpura as well as hemolytic uremic syndrome caused by Shiga toxin-producing *Escherichia coli*. Indeed, other forms of TMA may be due to distinct mechanisms that do not involve complement activation; for example, thrombotic thrombocytopenic purpura, diacylglycerol kinase epsilon pathogenic variants, and methylmalonic aciduria and homocystinuria cblC complementation type do not respond to treatment with the anti-C5 monoclonal antibody eculizumab.^24^^,^^25^ Furthermore, other diseases, such as systemic lupus erythematosus or sepsis, may have clinical and laboratory findings suggesting a diagnosis of TMA. Therefore, one of the challenges of clinical trials involving patients with aHUS is that there is a risk of patients without complement-mediated aHUS being enrolled, which could lead to either a poor response to treatment or spontaneous resolution (“response”) despite inappropriate treatment.^26^, ^27^, ^28^

Iptacopan (LNP023) is an oral, first-in-class, highly potent proximal complement inhibitor that specifically binds FB and inhibits the AP. In a first-in-human study, 80% or greater inhibition of the AP activity was achieved 2 hours postdose for subjects receiving iptacopan at 25 mg or higher doses.^29^ Inhibition of complement FB prevents activity of AP-related C3 convertase and the subsequent formation of C5 convertase and MAC (Figure 1). The advantage of this mechanism is that iptacopan also blocks amplification of both the classical pathway-dependent and the lectin pathway-dependent C5 activation while not blocking the generation of MAC initiated by the classical pathway and the lectin pathway. This is important because it means that MAC-dependent killing of *Neisseria* spp. through activation of the classical pathway will be maintained in immunized individuals.^13^ Moreover, iptacopan may allow for a more effective immune response to meningococcal infection in vaccinated individuals than anti-C5 antibody therapy.^30^ The well-established role of AP dysregulation in aHUS pathophysiology, the positive preliminary results with iptacopan in patients with IgA nephropathy,^31^ C3 glomerulopathy^32^^,^^33^ and paroxysmal nocturnal hemoglobinuria^12^ in phase 2 studies, coupled with the efficacy of approved complement inhibitor therapies in aHUS, provide a strong rationale to evaluate iptacopan directly in a phase 3 study for patients with aHUS.

Here, we describe the rationale and design of the pivotal APPELHUS phase 3 study, which aims to evaluate the efficacy and safety of iptacopan in adult patients with aHUS.

## Methods

### Study Population

Approximately 50 patients naïve to complement inhibitors (including anti-C5 antibody therapy) will be enrolled. All patients are to provide written consent and fulfill all the inclusion criteria and not meet any of the exclusion criteria (Table 1).

**Table 1:** Key inclusion and exclusion criteria

Inclusion	Exclusion
Aged ≥18 yrs	Treatment with complement inhibitors, including anti-C5 antibody
Evidence of TMA, including thrombocytopenia, evidence of hemolysis, and acute worsening of kidney function	ADAMTS13 deficiency (<10% activity)
	Shiga toxin-related HUS
	Positive direct Coombs test
Vaccination against *Neisseria meningitidis, Streptococcus pneumoniae* and *Haemophilus influenzae*	Known diacylglycerol kinase mediated HUS
	Identified drug exposure-related HUS
	HUS related to known genetic defects of cobalamin C metabolism
Patients with a kidney transplant are permitted; however, must: (a) have a known history of aHUS before current kidney transplantation, or (b) have no known history of aHUS, and persistent evidence of TMA at least 4 days after modifying the immunosuppressive regimen	Receiving plasma exchange/plasma infusion for ≥28 days before the start of screening for the current TMA
	Bone marrow/hematopoietic stem cell transplantation, heart, lung, small bowel, pancreas, or liver transplantation
	Patients with sepsis, severe systemic infection, COVID-19 infection, systemic infection which confounds an accurate diagnosis or impedes management of aHUS,
	Patients with a history of recurrent invasive infections caused by encapsulated bacteria
	Systemic sclerosis, systemic lupus erythematosus or antiphospholipid antibody positivity or syndrome, or any other autoimmune disease associated with HUS
	Chronic hemodialysis or peritoneal dialysis

ADAMTS13, a disintegrin and metalloproteinase with a thrombospondin type 1 motif, member 13; HUS, hemolytic uremic syndrome; TMA, thrombotic microangiopathy.

Other protocol-defined inclusion/exclusion criteria may apply.

Patients with aHUS reaching kidney failure that requires a transplant have high rates of disease recurrence posttransplant, with the outcome being worse in patients with *CFH, CFB,* and *C3* mutations.^34^^,^^35^ This study will aim for approximately 5 patients who have reached end-stage renal failure and undergone prior kidney transplantation to investigate iptacopan in patients post-transplant with relapsing disease. This should exclude patients with drug-associated TMA.

Diagnosis of aHUS is by exclusion and requires a differential diagnosis workup. Because the study is running in multiple centers worldwide where practice may differ, a patient selection committee has been established to review individual patient eligibility and confirm enrollment into the study. The committee will ensure an independent review of the aHUS diagnosis of each patient in this global study, thereby standardizing any geographic differences which may exist in diagnosing primary aHUS.

### Study Design

**Figure 2:**
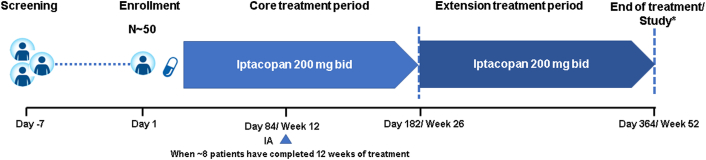
Study design aHUS phase 3—APPELHUS (NCT04889430). ∗At study completion, patients have the option to roll over into an open-label extension study. aHUS, atypical hemolytic uremic syndrome; bid, twice daily; IA, interim analysis; *N*, number of patients.

**Figure 3:**
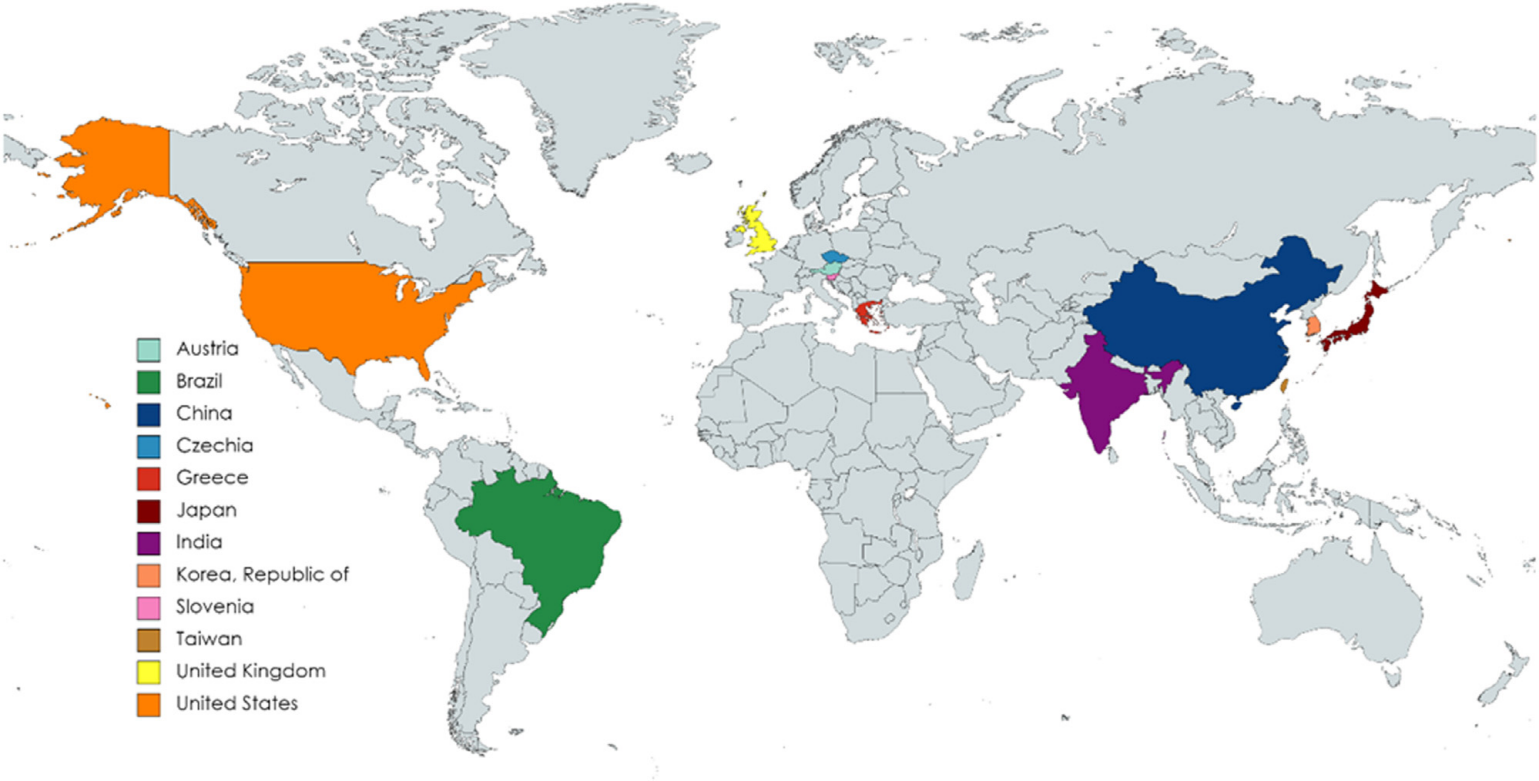
Multicenter recruitment sites involved in the aHUS Phase 3 APPELHUS study (NCT04889430). APPELHUS is currently recruiting patients in 26 centers across 12 countries.

APPELHUS (ClinicalTrials.gov Identifier NCT04889430) is a multicenter, single-arm, open-label study to demonstrate the efficacy and safety of iptacopan at a dose of 200 mg twice daily in adult patients with aHUS who are naïve to complement inhibitor therapy (including anti-C5 antibody therapy; Figure 2). At the time of writing, 26 centers are open for patient recruitment in 12 countries with more sites getting ready to open globally (Figure 3).

The study comprises a screening period lasting up to 7 days during which patients can receive plasma exchange or plasma infusion therapy, followed by a core treatment period where eligible patients will receive iptacopan 200 mg twice daily for 26 weeks. Patients will thereafter continue receiving iptacopan at 200 mg twice daily for an additional 26 weeks as part of the extension period. Patients completing the full 52 weeks and benefiting from treatment as judged by the investigator may be offered posttrial access to iptacopan by participating in an open-label extension study. A summary of key study assessments is provided in Table 2.

**Table 2:** Key study assessments

Assessment category	Assessment
Key efficacy assessments	Complete TMA response^a^
	Hematologic parameters
	eGFR and CKD stage
	Patient-reported outcomes (FACIT-fatigue)
Key safety assessments	Vital signs
	Laboratory evaluations in blood and urine
	Electrocardiogram
	Pregnancy and assessments of fertility
	Selected biomarkers related to disease progression
	Safety and efficacy parameters in patients with different aHUS genetic mutations
Other assessments	Health care resource utilization
	The need for hemodialysis or peritoneal dialysis will be monitored by the investigator during the study

aHUS, atypical hemolytic uremic syndrome; CKD, chronic kidney disease; eGFR, estimated glomerular filtration rate; FACIT-Fatigue, Functional Assessment of Chronic Illness Therapy-Fatigue; TMA, thrombotic microangiopathy.

a Complete TMA response is defined as: (1) hematological normalization in platelet count (platelet count ≥150 × 10^9^/l) and lactate dehydrogenase (below upper limit of normal); and (2) improvement in kidney function (≥25% serum creatinine reduction from baseline), maintained for 2 measurements obtained at least 4 weeks apart, and any measurement in between.

### Study Objectives

The primary objective is to assess the impact of iptacopan on TMA response following 26 weeks of treatment. Complete TMA response is a well-defined and accepted end point in clinical trials with aHUS and has been used in most recent clinical studies in patients with aHUS.^27^^,^^36^ The key objectives and endpoints are reported in Table 3.

**Table 3:** Key study objectives and related endpoints

Assessment	Objective	End point
Primary objective	To assess the proportion of patients treated with iptacopan achieving complete TMA response^a^ during 26 weeks of study treatment	Complete TMA response^a^ without the use of PE/PI or anti-C5 antibody therapy during 26 weeks of study treatment
Key secondary objectives	To assess the effect of iptacopan on time to complete TMA response	Time to achieve TMA response during 26 weeks of study treatment
	To assess the proportion of patients achieving an increase of ≥2 g/dl from baseline in hemoglobin levels	An increase in hemoglobin of ≥2 g/dl from baseline during 26 weeks of study treatment
	To assess the following at week 26: • The effect of iptacopan on hematological parameters (platelets, lactate dehydrogenase, hemoglobin) • The effect of iptacopan on eGFR, CKD stage, patient-reported overall fatigue severity, and health-related quality of life	Change from baseline in relevant parameters at week 26
	To assess the effect of iptacopan on dialysis requirement status	Proportion of patients on dialysis who no longer require it through 26 weeks of study treatment
	To assess the safety and tolerability of iptacopan	Adverse events/serious adverse events, laboratory parameters, and vital signs

CKD, chronic kidney disease; eGFR, estimated glomerular filtration rate; PE/PI, plasma exchange/plasma infusion; TMA, thrombotic microangiopathy.

a Complete TMA response: see Table 2 for definition.

### Statistical Considerations

#### Primary Efficacy Estimand

The primary analysis of the primary end point is the assessment of the proportion of patients reaching the status of complete TMA response over 26 weeks (core treatment period). The calculated TMA response rate will be compared with a threshold of 30% using a 2-sided 95% confidence interval (CI) for the proportion of complete TMA responders in iptacopan-treated patients based on asymptotic Gaussian approximation with continuity correction method. The 30% threshold was chosen based on 2 historical trials that are comparable in study design, population, and efficacy endpoints (eculizumab,^36^ ravulizumab^27^). The TMA response rates (and 95% CI) based on asymptotic Gaussian approximation with continuity correction method for the eculizumab and ravulizumab trials were 56.1% (39.7%, 72.5%) and 53.6% (39.6%, 67.5%), respectively. Given the single-arm nature of historical trials, it is difficult to hypothesize the actual extent of eculizumab or ravulizumab effect versus placebo. However, the lower boundaries of the 95% CI (∼40%) could be considered as a demonstrated effect over placebo and taken as the reference. A 30% threshold has been chosen to ensure the preservation of approximately 75% of this reference. A lower bound of the CI of ≥30% will demonstrate that iptacopan preserves a significant proportion of the treatment effect compared to that observed with anti-C5 antibody therapies eculizumab and ravulizumab. The primary analysis will account for different intercurrent events as explained in the following:

• Dialysis: if a patient requires dialysis during the 26-week treatment period, creatinine values during dialysis (from first day of dialysis through 5 days after the end of dialysis) will be excluded from the analyses. If a patient is on dialysis throughout the 26 weeks where baseline or postbaseline creatinine values are not available for determining the kidney improvement component of complete TMA response, the patient will be considered a nonresponder. For a patient on dialysis on day 1 (or within 5 days prior), baseline creatinine will be the first assessment ≥6 days postdialysis.
• Transfusion: platelet values obtained from the day of a platelet transfusion through 3 days after the transfusion will be excluded from the analyses.
• Discontinuation of study treatment for any reason: all available efficacy data will be included to calculate TMA response status without imputation.
• Plasma exchange/plasma infusion or anti-C5 antibody therapy use during the treatment period: patients will be considered as nonresponders.

The primary analysis will be performed on the full analysis set, which comprises all patients with aHUS (whose eligibility has been confirmed by the patient selection committee) to whom study treatment has been assigned and who have received at least 1 dose of study treatment. The patients discontinued from the study for not meeting the eligibility criteria based on central laboratory or not confirmed to be eligible by the patient selection committee will be excluded from the full analysis set.

#### Interim Analysis

An interim analysis will be performed when approximately 8 patients have completed 12 weeks of study treatment (day 84 visit). The intent of the interim analysis is to provide preliminary evidence on the efficacy and safety of iptacopan in treatment-naïve patients with aHUS. The interim analysis will include analysis of the primary end point (complete TMA response) at 12 weeks and its components (hematological normalization [platelet count and lactate dehydrogenase]), improvement in kidney function ([≥25% serum creatinine reduction from baseline]) as well as hematological parameters (platelets, lactate dehydrogenase, hemoglobin) and kidney outcomes (glomerular filtration rate and dialysis requirement) relevant to clinical benefit in patients with aHUS.

#### Safety Analyses

Patient safety will be closely monitored by investigators at prescreening, on days 1, 7, and 14, then at biweekly visits until 26 weeks, and monthly visits thereafter. At each visit, physical examination, vital signs (blood pressure, heart rate, pulse rate, respiratory rate, pulse oximetry), and body temperature will be assessed. Electrocardiograms will be recorded at selected visits and laboratory data will be measured at all visits.

Treatment-emergent adverse events, death, serious adverse events, and other significant adverse events, including those leading to treatment or study discontinuation will be summarized by primary system organ class and preferred term.

The use of prespecified rescue medications are not included in the protocol and will be selected at the discretion of the investigator based on local guidelines and availability.

#### Biomarkers

Blood and urine samples will be collected on days 1, 28, and 182; and analyzed for Wieslab AP, Factor Bb fragments and sC5b-9 at each of the 3 visits. These results will be used to determine if baseline levels change with treatment and predict patient outcomes and/or renal disease progression.

#### Sample Size Determination

The proposed sample size of 50 patients is sufficient to achieve a target absolute margin of error not larger than 0.15 (half-width of a 2-sided 95% CI for the proportion of patients reaching the status of responder) based on asymptotic Gaussian approximation with continuity correction method.

Under the assumption of the true TMA response rate for patients treated with iptacopan being 50%, at 1-sided alpha of 2.5%, the sample size of 50 will provide >80% probability that 2-sided 95% CI will exclude TMA response rate of 30%.

## Discussion

APPELHUS is a pivotal phase 3 study designed to evaluate the potential benefit of an oral, small molecule inhibitor of FB in patients with aHUS. Before the availability of anti-C5 antibody therapies, 56% of adults with aHUS progressed to end-stage kidney disease within the first year of disease onset regardless of genetic background.^8^ Despite the demonstrated efficacy of the anti-C5 monoclonal antibodies eculizumab and ravulizumab, they must be given via intravenous or subcutaneous routes, which is burdensome for patients, care givers, and the health care system. These therapies are also very expensive and are thus not available to all patients. Finally, anti-C5 antibody therapy dramatically increases the risk of meningococcal infection.^20^, ^21^, ^22^ Therefore, there is an unmet need for safer, more convenient treatment options.

APPELHUS is designed to assess the efficacy and safety of oral, twice-daily iptacopan in adult patients with aHUS who are naïve to complement inhibitor therapy, including anti-C5 antibody therapy. A single-arm design has been chosen for this study for the following reasons: (i) a placebo-controlled design is deemed unethical because aHUS is a severe, rapidly progressing disease requiring early treatment; even in countries where SoC (eculizumab or ravulizumab) is available, a placebo arm would not be ethical; (ii) single-arm, open-label designs are widely used in rare diseases because of challenges with recruitment and sample size; and (iii) a direct comparison with eculizumab is not possible due the large number of patients needed to appropriately power such a study in an ultrarare disease. All previously approved therapies for aHUS employed this design in their pivotal studies, although for eculizumab there was no commercially approved alternative.^27^^,^^36^ Ravulizumab received approval despite the absence of a direct comparison to eculizumab in aHUS, with both the adult and pediatric studies being single-arm in design.^26^, ^27^, ^28^^,^^37^ Iptacopan at 200 mg twice daily has been selected for this study based on the safety, efficacy, and favorable benefit-risk ratio data from the first-in-human studies and the phase 2 studies in C3 glomerulopathy,^32^^,^^33^ paroxysmal nocturnal hemoglobinuria ^12^ and IgAN.^31^ Because of the strength of evidence on the efficacy of iptacopan in the phase 2 studies of related indications (C3 glomerulopathy and paroxysmal nocturnal hemoglobinuria), we chose to assess iptacopan in aHUS directly in a phase 3 study. Therefore, this phase 3 study will be the first evidence of iptacopan efficacy specifically in aHUS.

A novel aspect of the study design is the patient selection committee; the patient selection committee was created for this study to avoid some of the pitfalls of recent clinical trials in aHUS.^27^ Misdiagnosis may occur in complement-mediated aHUS because of patients having other etiologies of TMA, various other genetic disorders, or having diseases such as sepsis or systemic lupus erythematosus that may have similar clinical and laboratory findings. Given the absence of a definitive diagnostic test available at study entry, there have been difficulties in making the diagnosis of aHUS, as illustrated by the recent ravulizumab trials.^27^^,^^38^ Patients in the initial eculizumab trial had a high mutation-positive rate (suggesting a high proportion of complement-mediated aHUS) whereas those in the ravulizumab trial had a low mutation rate, with an increased number of deaths (4 of 58 patients), and a number of patients enrolled who did not appear to have complement-mediated aHUS, and thus are not expected to respond to complement inhibitors.^26^, ^27^, ^28^ Enrolling patients without complement-mediated aHUS can lead to nonresponse to therapy or be a safety concern by administering a drug that is not appropriate for the patient.

Defective complement control on the endothelial surface results in a prothrombotic state in aHUS^2^ with loss and/or gain of function mutations in complement regulatory genes resulting in AP overactivation and impaired C3b degradation. The establishment of the key role of the AP in aHUS pathophysiology highlighted the rationale for complement inhibition as a therapeutic strategy and has resulted in the investigation of several novel targeted agents, such as iptacopan. Whereas most complement therapies target the terminal pathway, iptacopan inhibits the proximal node of the AP, which plays a key role in the amplification of the classical and lectin pathways.^12^^,^^14^

The anti-C5 antibody therapies, eculizumab and ravulizumab, inhibit the complement system at the terminal step after convergence of the classical, lectin, and AP by preventing cleavage of C5 to C5a and C5b and formation of the terminal complement complex C5b-9 (or MAC).^18^ Iptacopan targets FB, an AP-specific serine protease that complexes with C3b to drive the catalytic activity of the AP C3 and C5 convertases. Benefits to the selective inhibition of FB include suppression of the activity of AP-related C3 convertase, the initial and main driver of the disease, thus blocking the cleavage of C3 and activation of the amplification loop. In turn, this blocks downstream generation of the AP-related C5 convertase complex, and formation of C5a anaphylatoxins and MAC from the AP, preventing excessive complement deposition and tissue damage. Iptacopan offers a unique advantage over current SoC anti-C5 antibody therapies (eculizumab or ravulizumab), which are associated with a high risk of infection by encapsulated bacteria, because it does not fully block the generation of MAC initiated by the classical and lectin complement pathways, and thus infection risk should theoretically be reduced. However, further study of this aspect is needed. *Ex vivo* studies of sera from vaccinated individuals suggest that AP inhibition with iptacopan does not increase susceptibility to meningococci and pneumococci infection.^30^^,^^39^ In contrast, inhibiting downstream complement components, such as C5, causes an impaired response to infection in sera from vaccinated patients.^33^ To date, iptacopan has demonstrated a favorable safety profile and was well-tolerated in phase 1 and phase 2 clinical studies, which required vaccination.^12^^,^^32^^,^^33^ Importantly, no serious infections caused by encapsulated bacteria were reported in these studies. These findings suggest that iptacopan may have a lower risk of meningococcal infection than anti-C5 antibody therapy, although vaccination remains essential and is required for patients in APPELHUS.

Eculizumab and ravulizumab are both FDA-approved and EMA-approved for the treatment of aHUS^22^^,^^40^ and paroxysmal nocturnal hemoglobinuria.^22^^,^^41^^,^^42^ Both eculizumab and ravulizumab are administered via injection, which puts a burden on patients and health care systems, and is a particular disadvantage for administration to young patients with aHUS. Iptacopan has the advantage of being an oral formulation which, given the high pediatric rate of aHUS, is important. For instance, oral treatment would remove the need for long-term indwelling catheters in young children. Major advantages of oral over intravenous and subcutaneous route are the absence of cannula-related infections, lower costs involved in equipment and administration, ability to self-administer, and improved quality of life given the absence of injections and decreased travel time to health care centers for infusion administration. It is therefore hoped that iptacopan, as an oral therapy, will reduce the burden on both patients and health care systems.

Although the introduction of eculizumab, and later ravulizumab, undoubtedly transformed the natural history of aHUS, there remain unmet needs and unanswered questions regarding existing therapies^3^^,^^16^^,^^18^ surrounding the risk of infection (meningitis) resulting from complement inhibition^12^^,^^13^ and the need for repeated infusions.^43^^,^^44^ Iptacopan has the potential to become an effective and safe treatment for aHUS with a lower treatment burden because of oral administration. Given its central role in disease pathogenesis, inhibiting the initial driver of the disease, AP dysregulation, by targeting complement FB, it is an attractive therapeutic strategy to ameliorate aHUS disease progression.^9^ This phase 3 APPELHUS study is the first study of iptacopan in aHUS and will attempt to overcome the challenge of enrolling the correct patients seen in prior studies of aHUS by having a centralized patient selection committee.

## Disclosure

DK reports grant support from Medical Research Council, Wellcome Trust, Kidney Research UK, Complement UK, Fight For Sight, and Macular Society; and is a consultant for Silence Therapeutics, Alexion Pharmaceuticals, Novartis, Apellis, and Sarepta. LAG is a consultant for Novartis, Alexion, Roche, Apellis, and Alnylam.

RGK, CWC, and AC are employees of Novartis Pharma, East Hanover, NJ, USA. SV Is an employee of Novartis Healthcare, Hyderabad, India. MD is an employee of Novartis Pharma AG, Basel, Switzerland. FF has received consultancy and/or speaker honoraria from Roche, Alexion, Apellis, Achillion, Novartis, and Alnylam. AB declares no conflicts of interest.
